# Can Sediments Contaminated by Mining be a Source of Mercury in the Coastal Environment Due to Dredging? Evidence from Thermo-Desorption and Chemical Speciation

**DOI:** 10.1007/s00128-021-03159-x

**Published:** 2021-03-02

**Authors:** Stefano Covelli, Elisa Petranich, Elena Pavoni, Sergio Signore

**Affiliations:** 1 Dipartimento Di Matematica E Geoscienze, Università Degli Studi Di Trieste, Via Weiss 2, 34128 Trieste, Italy; 2 Dipartimento Di Scienze Chimiche E Farmaceutiche, Università Degli Studi Di Trieste, Via Giorgieri 1, 34127 Trieste, Italy; 3 Autorita’ di Sistema Portuale del Mare Adriatico Orientale - Porto di Trieste, via Karl Ludwig von Bruck, 3, 34144 Trieste, Italy

**Keywords:** Sediments, Dredging, Mercury, Chemical speciation, Bioavailability

## Abstract

The sediments in the Gulf of Trieste (northern Adriatic Sea, Italy) are contaminated by mercury (Hg) due to historic mining which took place in Idrija (Slovenia). Despite many studies having been done regarding Hg, no information is available on the potential impact of dredging required along the main channel approaching the Port of Monfalcone. Sixteen surface sediment samples were collected along the channel to determine both total Hg concentration and chemical species using the thermo-desorption (TD) technique. Six samples were also chosen to apply a selective sequential extraction (SSE). The TD technique showed the maximum Hg release approximately at 260 and 335°C, corresponding to metacinnabar (β-HgS) and cinnabar (α-HgS), respectively. The SSE demonstrated that Hg was mainly associated with poorly soluble or insoluble compounds (98.7%). A resuspension event over a limited period of time can be considered of negligible impact to the water column due to the scarce Hg mobility from sediments.

Most ports are usually important sites of industrial and urban activities and are recognised as potential reservoirs for organic and inorganic contaminants which, due to poor environmental management, accumulate in the bottom sediments (e.g. Schintu et al. 2016). Sediments can be a secondary source of contamination due to both desorption of the labile components (Caplat et al. 2015), remobilisation (Kelderman and Osman 2007) and resuspension events (Gibson et al. 2015; García-Ordiales et al. 2020) as a result of changes in the physico-chemical conditions. For instance, dredging to ensure safe navigation can modify the original physico-chemical features of sediments due to their partial resuspension in the water column (Fisher et al. 2015), also having negative effects on the benthic biota (e.g. Moog et al. 2018). The eventual desorption of chemical species from the solid phase can lead to the formation of more mobile, reactive and toxic chemical forms, potentially bioavailable to the aquatic trophic chain (Schneider et al. 2018).

Increasing concern about the contamination of sediments has resulted in European as well as national regulations enforcing severe control and monitoring of contaminated sediment movements. There is also a continuing debate on the possible harm that the physical resuspension of contaminated sediments might cause to the trophic chain and marine activities such as mussel and fish farming (e.g. Bocchetti et al. 2008).

The marine coastal environments of the northern Adriatic Sea (Italy) have been widely investigated in relation to the historical mercury (Hg) contamination associated with the Idrija (NW Slovenia) mining activity which was in operation for 500 years (Covelli et al. 2001). Contamination is due to the fluvial inputs of the Isonzo/Soča River system discharging into the Gulf of Trieste (Faganeli et al. 2003), a shallow semi-enclosed basin (A = 500 km^2^) with a maximum water depth of 25 m (Fig. 1). High Hg concentrations both in sediments (0.10–23.30 mg kg^−1^; Covelli et al. 2001) and in the suspended particulate matter (PHg, 0.20–2.70 mg kg^−1^; Covelli et al. 2007) were also found to be two orders of magnitude higher than the natural background (0.13 ± 0.04 mg kg^−1^ (Covelli et al. 2006). Moreover, investigations on Hg mobility at the sediment–water interface were performed (Emili et al. 2014, 2016), in particular when hypoxic-anoxic conditions (Ullrich et al. 2001) promote inorganic Hg transformation into methylmercury (MeHg), the most toxic organic chemical form easily bioaccumulated by biota. Several studies on Hg bioaccumulation in the aquatic trophic chain have also been done in this coastal environment, since aquaculture activities such as clam harvesting and mussel and fish farming are common (e.g. Giani et al. 2012; Petranich et al. 2018).

**Fig. 1:**
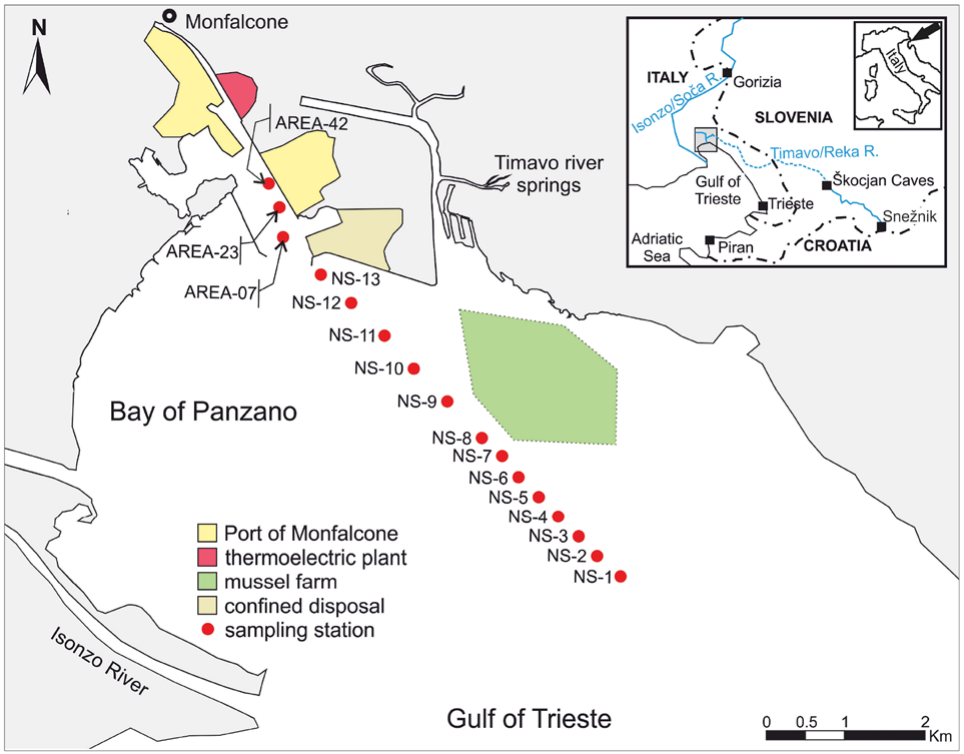
Map of the study area and location of the sampling sites along the main axis of the channel approaching the Port of Monfalcone in the Gulf of Trieste

In the present study, 16 sediment samples were collected from the main channel approaching the Port of Monfalcone (Fig. 1), one of the northernmost ports of the Adriatic Sea in the Gulf of Trieste, to evaluate the potential release of Hg from sediment to the water column due to resuspension caused by dredging. The aim was to identify the Hg chemical forms present in the sediments by applying two different speciation approaches: (1) the thermo-desorption (TD) technique which is based on the real-time detection of Hg released from the sediment matrix during gradual heating (Mashyanov et al. 2017); (2) the Selective Sequential Extraction (SSE) procedure (Bloom et al. 2003), which applies five extracting solutions able to recover different Hg species from the most labile (e.g. HgCl_2_) to the most stable form (α-HgS).

## Materials and Methods

The Bay of Panzano (Fig. 1), located in the north-eastern sector of the Gulf of Trieste, is a shallow and sheltered embayment prone to the accumulation of fine sediments. The Port of Monfalcone, situated between the Isonzo River mouth to the south and a mussel farming area to the west, is connected to the open sea by a main access channel for which dredging is planned in the near future to allow for the navigation of cargo with a high draft.

Sediment cores were collected by a scuba diver by hand-pushing an inhouse modified Shelby corer (96 cm i.d., 200 mm length) into the sediment from 16 sites (Fig. 1) along the main axis of the channel approaching the Port of Monfalcone. The sediment cores were extruded on board and the first 20 cm of the samples taken were homogenised, collected in glass containers and stored in a cool, dark room (~ 4°C) before total Hg (THg) concentration and speciation analyses and grain-size determination.

For grain-size analyses, approximately 20 g of fresh sediment were treated with H_2_O_2_ (10%) for a 24 h period to eliminate most of the organic matter. Subsequently, the sediment was wet-sieved through a 2 mm sieve to remove coarse shelly fragments. The resulting < 2 mm fraction was analysed using a laser granulometer (Malvern Mastersizer mod. 2000).

Total Hg in the dry sediment was determined using the Direct Mercury Analyser (DMA-80, Milestone) according to the EPA Method 7473. The limit of detection (lod), expressed as the amount of Hg content in a sample, was approximately 0.005 ng. Samples were analysed in triplicate and the results were compared with the values obtained on the basis of three replicates (2.81 ± 0.15 mg kg^−1^ Hg) of a certified reference material (2.98 ± 0.36 mg kg^−1^; PACS-3 Marine Sediment CRM, NRCC, Canada), and the relative standard deviation of at least three determinations was < 2%.

Each Hg species in a solid matrix interacts in different ways, exhibiting a varying degree of solubility and mobility (Rumayor et al. 2013). The TD technique, the so-called *thermoscanning technique*, represents the powerful and rapid application for the direct analysis of Hg species in solid samples. This technique is based on the real-time detection of Hg release from a sample during gradual heating (Mashyanov et al. 2017), where each Hg species is released from the matrix according to its desorption temperature (Rumayor et al. 2013).

In this study, the RA-915 Mercury analyser coupled to a Pyro-915 + furnace (Lumex Instruments) were used to thermoscan all the samples. Approximately 70–80 mg of sample was placed into the quartz boat of a thermocouple and inserted into the furnace where the temperature was gradually increased from ambient to 710°C (0.5°C s^−1^). The thermocouple was used to continuously monitor the temperature increase. Quantification was carried out by peak integration using the RAPID software. The set was calibrated with the SBPS-3 reference material (310 µg kg^−1^) and precision and accuracy were determined using a sediment standard reference material (PACS-3). To detect Hg species in the samples, standard Hg compounds such as cinnabar (α-HgS, from the Idrija mine), meta-cinnabar (β-HgS) and mercury oxide (HgO red), which was the result of extremely high concentrations of Hg, were mixed with synthetic CaCO_3_ and then desorbed. The choice of the synthetic CaCO_3_ is due to the mineralogical characteristics of the sediments which are rich in carbonates (Brambati 1970).

The SSE procedure was performed on six sediment samples (NS-3, NS-4, NS-5, NS-6, NS-7 and NS-8) representative of the whole sample set. These samples were chosen as they were taken from a location close to the mussel farm which is considered the main recipient of the potential effects induced by the resuspension of Hg in the water column. In addition, according to the high total Hg concentration, these sediment samples also exceeded the contamination threshold limit for soils in sites intended for commercial and industrial use (5 mg kg^−1^) established by Italian Legislative Decree n. 152/06. This is of relevant concern since 5 mg kg^−1^ is the threshold limit in case the proposed purpose is to confine dredged sediments to a coastal disposal site (Fig. 1) planning their re-use without any physical or chemical treatment of remediation. One possible destination for these sediments, in fact, is their relocation to the harbour area to enlarge the dock area.

The five-step procedure described by Bloom et al. (2003), and subsequently adapted for sediments by Shi et al. (2005), consists of five reagent solutions with increasing extracting capacity (Fig. 3). After the last step, the solid residue was air-dried and analysed with DMA-80. The analysis of the five extracted solutions was performed by applying the Cold Vapour-Atomic Fluorescence Spectrometry (CV-AFS) technique. The method was verified for all samples by summing the ratio of the extracted Hg in each phase (Hg_x_) to THg in the sample (ΣHg_x_/THg).

## Results and Discussion

According to Shepard’s (1954) textural classification, surface sediments are dominated by silt (67.8%–82.5%), followed by sand (0.4%–15.7%) and clay (11.6%–28.8%) (Table 1). The surface sediments displayed a variability of two orders of magnitude for THg concentrations: from 0.30 mg kg^−1^ (NS-12) to 13.5 mg kg^−1^ (NS-5), showing a decreasing trend from the offshore area to the innermost sector of the port (Table 1). High concentrations of trace elements in sediments are generally associated with the fine sediment fractions such as silt and clay (Acquavita et al. 2012a). This peculiarity is mainly due to physico-chemical factors that favour the element retention capacity of the finest particles, namely silt and clay (Bengston and Picado 2008): specific surface area, ion-exchange capacity, surface electric charges (as clay minerals), organic matter content and the occurrence of Fe and Mn oxy-hydroxides (Feyte et al. 2010). However, previous studies in the Gulf of Trieste have demonstrated that this element is distributed in association with all the grain-size fractions < 2 mm (Covelli et al. 2001). By applying the TD technique, Biester et al. (2000) also indicated that Hg in this area is mostly present in detrital form (cinnabar) in sandy-silty sediments near the Isonzo River mouth, whereas, in the more distant areas, it is in ionic form (as Hg^2+^) bonded to fine particles adsorbed onto clay minerals and/or partially associated with organic matter.

**Fig. 2:**
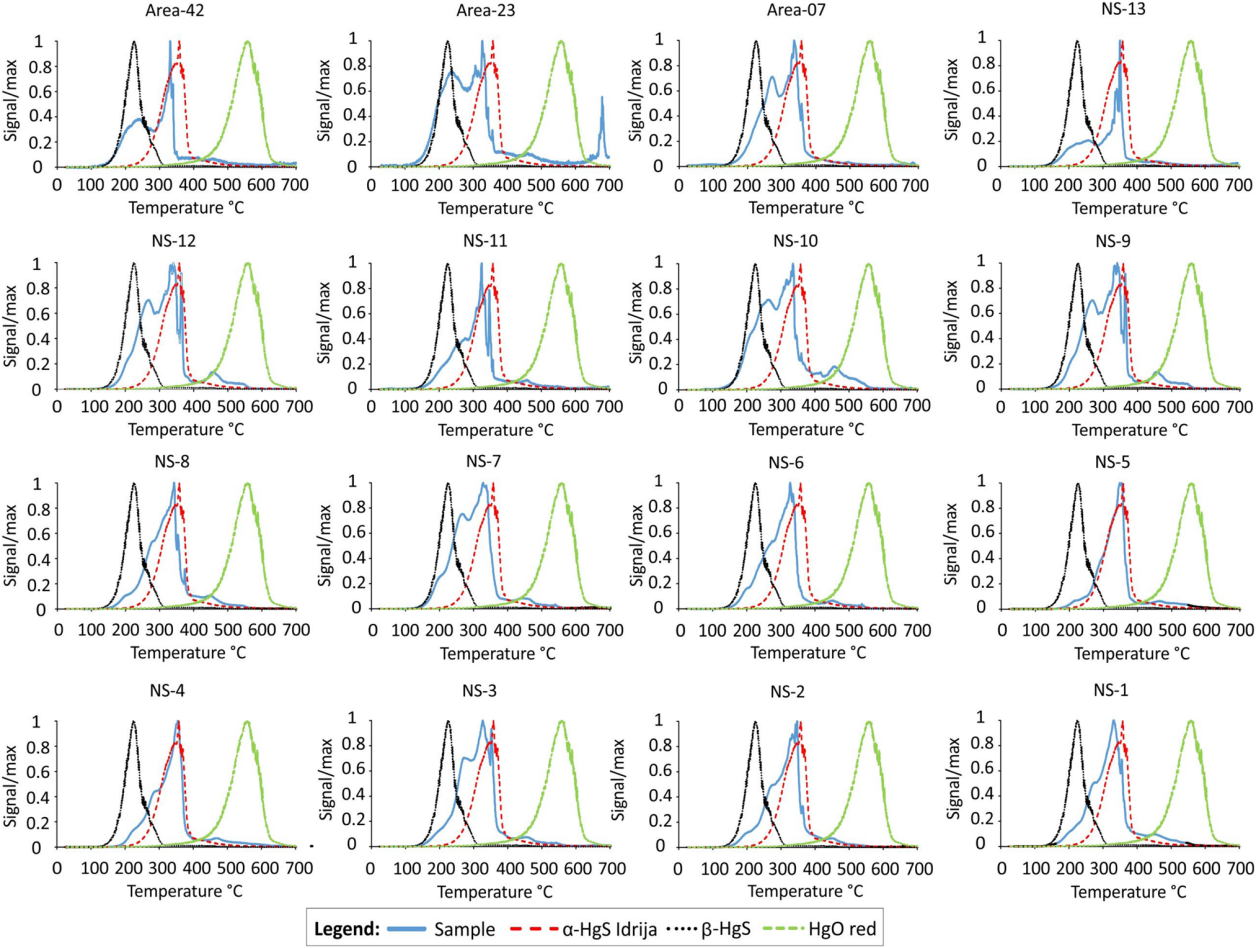
Thermoscanning (TD) curves obtained from sediment samples and standard Hg compounds

The results obtained using the TD technique applied to all the sediment samples showed three ranges of temperature peaks for Hg release: 252–275°C, 300–364°C and 454–475°C (Fig. 2). By comparing the TD curves obtained from each sediment sample with those of single standard Hg compounds, the Hg species occurring in the samples may be identified. Three different temperature peaks corresponding to the maximum release of Hg from the standard compounds were observed: 351 ± 12.7°C (α-HgS_Idrija_), 226 ± 8.0°C (β-HgS) and 559 ± 12.6°C (HgO red).

**Table 1:** Grain-size and THg concentrations in the sediment samples from the innermost sector of the channel to the offshore area

Sample	Sand (%) (2000–62.5 µm)	Silt (%) (62.5–2 µm)	Clay (%) (< 2 µm)	THg (mg kg^−1^)
AREA-42	15.7	72.7	11.6	0.54 ± 0.04
AREA-23	10.4	75.3	14.3	0.36 ± 0.04
AREA-07	0.6	76.2	23.2	2.81 ± 0.25
NS-13	8.1	76.4	15.4	0.58 ± 0.02
NS-12	7.0	71.6	21.4	0.30 ± 0.00
NS-11	3.2	77.0	19.8	1.00 ± 0.19
NS-10	1.9	73.7	24.4	0.94 ± 0.12
NS-9	2.4	72.8	24.8	1.12 ± 0.05
NS-8	7.8	82.5	9.7	6.36 ± 0.05
NS-7	3.0	72.4	24.6	8.65 ± 0.05
NS-6	3.4	67.8	28.8	8.34 ± 0.27
NS-5	0.4	73.3	26.3	13.5 ± 0.27
NS-4	0.5	71.6	27.9	11.1 ± 0.19
NS-3	0.4	77.9	21.7	9.01 ± 0.27
NS-2	0.6	72.3	27.1	7.32 ± 0.31
NS-1	0.5	71.4	28.1	8.47 ± 0.12

The release of Hg species weakly associated with the sediment mineral matrix generally occurs at temperatures lower than 250°C (Biester et al. 2000; Cavoura et al. 2019 and references therein), whilst higher desorption temperatures are required to release Hg species strongly associated with organic compounds such as humic acids or present as α-HgS (Biester et al. 2000).

The sediments sampled in the innermost sector of the port (Area-42, Area-23 and Area-07) showed TD curves characterised by two peaks: the first at 258°C and the second at 331°C, which could correspond to the two cinnabar species, β-HgS and α-HgS_Idrija_, respectively (Fig. 2). This difference in terms of desorption temperature between the two cinnabar species is related to the lower stability of β-HgS compared to α-HgS_Idrija_, for which less energy, and then lower temperature, is required to desorb Hg.

Rumayor et al. (2013) and Baptista-Salazar et al. (2017) also found the temperature peak of α-HgS in standard compounds at 305 ± 12°C and approximately at 330°C in the samples. A similar explanation can also be valid for the other sediment samples, where α-HgS and β-HgS correspond to the totality of the Hg present. Moreover, for some samples (NS-8, NS-9, NS-10 and NS-12), small Hg peaks between 400 and 500°C were detected (< 1.5% on THg), and attributed to HgO red, as suggested by Biester et al. (2000) and Sedlar et al. (2015), despite the TD curve of the standard compound showing the maximum peak at 559°C (Fig. 2). This discrepancy could be due to the characteristics of the sedimentary matrix which may interfere with the release of Hg, anticipating or delaying it with respect to the standard compound (Mashyanov et al. 2004).

**Fig. 3:**
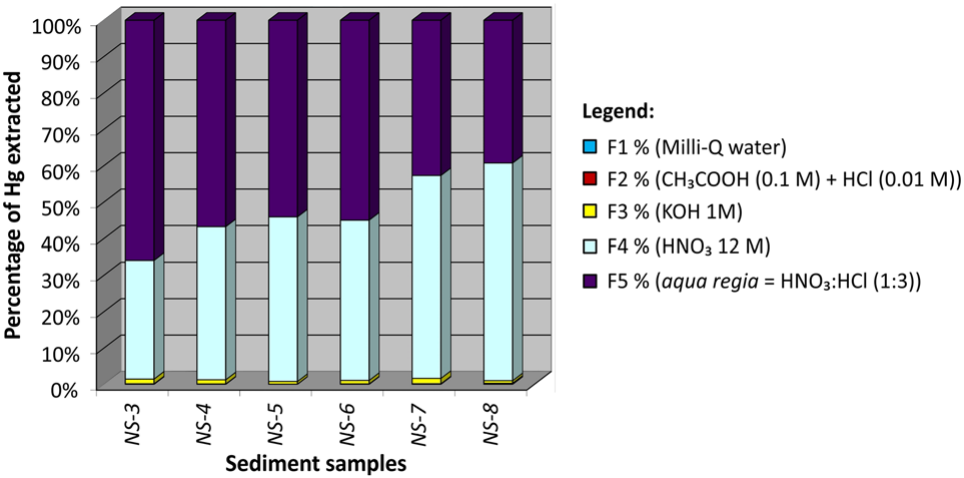
Mercury fractions (%) obtained by the SSE procedure performed on six samples

The SSE procedure allowed for the differentiating of Hg compounds in terms of dissimilar behaviour classes (Bloom et al. 2003). The F1 and F2 fractions, which comprise highly soluble and easily exchangeable compounds (i.e. HgCl_2_, HgSO_4_ and HgO) and are fully dissolved by the ‘‘*in vitro human stomach simulation*’’, showed very low percentages of Hg extracted (Fig. 3).

These two above-mentioned fractions are considered the main potential substrates for Hg methylation (Ullrich et al. 2001). However, the maximum value of the sum F1 + F2 for our samples is 0.36% (avg. 0.21%) which appears to limit the availability of inorganic Hg as substrate for methylation. If compared to Hg speciation studies performed on coastal sediments (Table 2), the maximum value obtained in the study area is higher than those found at the Isonzo river mouth (0.11%, Emili et al. 2014), the Grado Lagoon (0.22%, Covelli et al. 2011) and Guanabara Bay (0.11%, Covelli et al. 2012), where Hg was directly discharged into the aqueous media from a chlor-alkali plant (CAP). Comparable percentages were found in the Ravenna Lagoon (0.40%, Covelli et al. 2011), impacted by Hg from an acetaldehyde production plant where Hg was used as a catalyst, and in the Taranto Harbour (0.30%, Emili et al. 2016). Conversely, a notably higher percentage of F1 + F2 (11.65%) was found in the Aussa River sediments which received effluents from a CAP (Covelli et al. 2009). The results obtained from our study area for the two most mobile fractions does not constitute an issue of particular concern with respect to other coastal sediments contaminated by Hg.

**Table 2:** Partitioning of Hg in the solid phase according to the selective extraction procedure in selected costal sites

	n	Hg (mg kg^−1^)	Hg-w (%)	Hg-h (%)	Hg-o (%)	Hg-e (%)	Hg-s (%)
(1) This study	5	6.36–13.50	0.08–0.18	0.04–0.18	0.71–1.47	32.6–59.7	39.3–56.7
(2) Isonzo R. mouth (ITA)	1	13.27	0.08	0.03	0.77	23.45	75.68
(3) Grado Lagoon (ITA)	2	10.75–13.37	0.16–0.20	0.01–0.02	2.72–4.28	43.58–53.31	42.24–53.49
(4) Aussa River (ITA)	6	0.82–5.69	0.66–5.60	0.96–6.05	5.59–24.81	33.11–90.57	0–57.95
(5) Ravenna Lagoon (ITA)	2	14.40–19.10	0.20–0.38	0–0.02	4.77–6.32	87.00–87.81	5.67–7.83
(6) Taranto Harbour (ITA)	1	6.61	0.30	0.00	4.20	93.10	2.30
(7) Guanabara Bay (BRA)	6	1.54–3.22	0–0.02	0–0.09	1.11–6.69	92.85–98.65	0.15–1.46

Contamination is due to mining activities (1, 2 and 3), chlor-alkali plant (4,7), acetaldehyde factory (5) and Italian Navy shipyard-arsenal (6). From: (2) Emili et al. (2014); (3,5) Covelli et al. (2011); (4) Covelli et al. (2009); (6) Emili et al. (2016); (7) Covelli et al. (2012)

The F3 fraction, corresponding to the organo-chelated Hg fraction, bound to the organic substance (humic, fulvic and amino acids) with a moderate mobility, is also extremely low in terms of percentage (0.71%–1.47%, avg 1.06%). Bloom et al. (2003) stated that inorganic Hg extracted in the F3 fraction is mainly correlated with methylation potential. By adding the F3 fraction to the sum F1 + F2, it can be hypothesised that the three fractions may constitute the Hg pool for methylation. Particulate-bound Hg(II) may not have to be desorbed or dissolved in the aqueous phase to make it available for microbial uptake and methylation (Zhang et al. 2019). Dissolved organic matter (DOM) binds strongly with both Hg and MeHg in natural ecosystems and affects bioavailability depending on DOM composition since terrestrial DOM shows an inhibitory effect on MeHg uptake by bacteria and phytoplankton compared to marine DOM (Schartup et al. 2015). However, to assess the bioavailability of MeHg is complicated due to several processes that occur between the solid and liquid phases. MeHg is mainly produced in the surface sediments by anaerobic bacteria (Hsu-Kim et al. 2013). MeHg concentrations in aquatic systems vary widely and do not necessarily correlate with the total amount of mercury in water or sediments (Acquavita et al. 2012a; Covelli et al. 2001). In addition, it is the balance between methylation and demethylation, due to sunlight at the surface of the water column or in the sediments, which determines the amount of MeHg in an aquatic system (Hines et al. 2006). The highest percentage of F1 + F2 + F3 obtained for our samples (1.69%) compared to other Hg contaminated sediments (Table 2) is only higher than the Isonzo River mouth (0.88%) and than 3 out of 6 samples from Guanabara Bay. For the other sites (Table 2), the sum F1 + F2 + F3 is always higher compared to this work, especially for the Aussa River sediments. Overall, in spite of the total Hg concentrations in sediments, the three fractions with high mobility are found only in trace amounts.

Indeed, most of the extracted Hg (approximately 99%) was included in the last two fractions, distributed in a variable percentage between F4 and F5, depending on the sample (Fig. 3). With the exception of NS-7 and NS-8, the F5 fraction, which is characterised by a mineral bound fraction and includes α-HgS, β-HgS and Hg immobilised by pyrite (Huerta-Diaz and Morse 1992), prevails (> 50%). The F4 fraction represents, on average, 46% of the extracted Hg and constitutes the most abundant fraction in NS-7 (55.6%) and NS-8 (59.7%). This fraction includes the forms of Hg bound to strong complexes, mainly elemental Hg together with the Hg forms linked to amorphous organo-sulphides and to the crystalline phase with Fe and Mn oxides. These compounds are normally poorly soluble and not very bio-accessible, which can release significant amounts of Hg only in exceptional conditions of anoxia (Emili et al. 2014). Moreover, since elemental Hg was not detected in the TD analysis (maximum peak at 100°C), the F4 of the SSE should correspond to Hg forms linked to amorphous organo-sulphides and to the crystalline phase with Fe and Mn oxides. In agreement with Reis et al. (2015), indeed, our first peak at 258°C could also correspond to Hg bound to iron oxides which is released between 100 and 285°C rather than β-HgS. However, no standard for Hg associated with Fe oxides was available to verify this hypothesis.

In conclusion, Hg distribution in the different fractions obtained by SSE (Fig. 3) indicates that the element is strongly associated with the less mobile fractions. The TD technique performed on all the sediment samples confirmed that the main Hg forms in these sediments are not easily remobilisable. According to these results, we should not expect a large contribution of Hg released in its dissolved form due to the physical resuspension of bottom sediments. In other contexts, it was observed that metal release during sediment disturbance events tends to be higher under oxic conditions thus suggesting oxidative dissolution of sulphide mineral phases and oxidative degradation of organo-chelated Hg compounds which may contribute to high Hg concentrations (Gibson et al. 2015). Conversely, our results are in agreement with experimental observations obtained from simulating a resuspension event of bottom sediments (11.4 mg kg^−1^ total Hg and 7.5 ng g^−1^ MeHg) periodically subjected to dredging activities in the Grado Lagoon (Acquavita et al. 2012b). The experiment revealed that the release of Hg species from the solid to the dissolved phase became negligible quickly after the event. The concentrations of both inorganic Hg and MeHg found in the water column after resuspension were comparable to those monitored in situ in the Lagoon. This evidence supports the hypothesis that the effects of a resuspension event are temporary due to dilution of the Hg species in the water column and their settling back to the bottom in association with the sediment particles.

Results from this research demonstrated that both speciation techniques were found to be valid and comparable to each other to discriminate between mobile and non-mobile Hg chemical species from the sediment to the water column.
